# Access to movement disorders care and advanced surgical therapies in a tertiary care center

**DOI:** 10.3389/fneur.2026.1743834

**Published:** 2026-03-31

**Authors:** Jacob A. Alderete, Ashley Madera, Daniela Markovic, Ziad Rifi, Maya Harary, Katherine A. Fu, Katy A. Cross, Ausaf A. Bari, Adys Mendizabal

**Affiliations:** 1 Department of Neurosurgery, University of California Los Angeles, Los Angeles, CA, United States; 2 Department of Neurology, University of California Los Angeles, Los Angeles, CA, United States; 3 Department of Neurology, Barrow Neurological Institute, Phoenix, AZ, United States; 4 Division of General Internal Medicine and Health Sciences Research, University of California Los Angeles, Los Angeles, CA, United States; 5 Department of Neurosurgery, Mt. Sinai Health System, New York, NY, United States

**Keywords:** access to neurological care, deep brain stimulation, essential tremor, health disparities, health services, Parkinson’s disease

## Abstract

**Background:**

Barriers in access to Movement Disorders specialty care may explain sociodemographic differences in the utilization of deep brain stimulation (DBS) surgery for medically refractory Parkinson’s disease (PD) and essential tremor (ET).

**Methods:**

This retrospective chart review used electronic medical records between 2012 and 2022 from a single tertiary movement disorders center to characterize DBS surgery patterns among a diverse group of movement disorders patients. Patients were diagnosed with PD, ET, or unspecified tremor aged 18 or older. Patient characteristics were summarized and compared between those who did and did not undergo DBS implantation using chi-square tests for categorical variables and Wilcoxon rank-sum tests for continuous variables. Multivariable regression model was used to identify associations between clinical and sociodemographic covariates and DBS surgery.

**Results:**

Of 3,286 PD/ET patients between 2012 and 2022, 12.1% underwent DBS surgery. Those who underwent surgery were younger and had fewer medical comorbidities. Within the surgical group, there were higher proportions of Hispanic ethnicity, non-English language preference, Medicare or Medi-Cal insurance, and residence in neighborhoods with lower socioeconomic status (SES). On multivariable logistic regression analysis, MediCal insurance and low neighborhood SES were associated with higher odds of surgery. Conversely, younger age at initial visit, single marital status, and Asian race were associated with lower odds of surgery.

**Conclusion:**

Sociodemographic variables associated with higher odds of receiving DBS surgery included low SES and Medi-Cal insurance. This may reflect a referral bias suggestive of better access to our center for DBS surgery than for routine movement disorders care for those from low SES and Medi-Cal. Future studies within our center will focus on quantitative and qualitative evaluation of referral processes for the treatment of advanced movement disorders in order to better understand and improve accessibility of these therapies to all patients in our health system and nationally.

## Introduction

Care provided by movement disorders specialists has been shown to improve diagnostic accuracy, improve overall quality of life, and minimize delays in access to advanced therapies such as deep brain stimulation (DBS) (1, 2). Unfortunately, the limited number of movement disorders specialists in the United States is unable to meet the growing clinical demand leading to disparities in care across gender, racial, socioeconomic, and geographic lines. Though the use of DBS has shown an upward trend since its approval for both Parkinson’s disease (PD) and essential tremor (ET), underutilization in certain groups has persisted and is compounded by this underlying barrier to access specialized care (3). More specifically, women, people of color, those from low socioeconomic backgrounds, and rural patients are more likely to face challenges in accessing DBS (3–9). Beyond identifying trends in these domains, the interaction between access to movement disorders care and advanced movement disorders therapeutics such as DBS is of interest to better understand the systems-level factors and processes that may influence patient access to both of these.

The University of California Los Angeles (UCLA) Health System serves a highly diverse patient population in Los Angeles (LA) County. In LA County approximately 49% of all residents identify as Hispanic/Latino (hereafter, Hispanic), 16% identify as Asian, and approximately 55.1% of residents speak a language other than English at home (10). In terms of neighborhood socioeconomic status (SES), LA County falls within the 19th percentile in national ranks of Area Deprivation Index (ADI), an index that measures adverse social exposures ranging from 1 to 100, with higher ADI representing greatest deprivation and lowest SES (11, 12). Given the patient diversity in our center’s catchment area, we were interested in characterizing the movement disorders patient population at UCLA, DBS surgery completion rates, and clinical and sociodemographic factors associated with completion of DBS surgery within our center.

## Methods

This study was determined to be exempt by the UCLA Institutional Review Board at the University of California, Los Angeles.

Patients were identified from institutional electronic medical records between January 1, 2012 to December 15, 2022, age 18 or older, with a diagnosis of Parkinson’s disease, essential tremor, or unspecified tremor using ICD codes: ICD-9: 333.1, ICD-10-CM: G20, ICD-10-CM: G20*, ICD-10-CM: G25.0, ICD-10-CM: R25.1, ICD-10-CM: G25.2. Included patients were evaluated by Movement Disorders clinicians and further stratified by those with and without DBS implantation, using CPT codes 61863, 61864, 61867, 61868, 61885, 61886. Patient characteristics were summarized using medians (IQR) or means with standard deviations for continuous variables and frequencies with percentages for categorical variables. Comparisons between those who underwent DBS and those who did not were performed using chi-square tests for categorical variables and Wilcoxon rank-sum tests for continuous variables.

To assess associations between covariates and DBS surgery, we fitted logistic regression models. We first estimated unadjusted odds ratios (ORs) for each covariate in separate univariate models. A multivariable logistic regression model was then constructed, adjusting for all covariates including age at initial visit, sex, race/ethnicity, marital status, insurance type, comorbidity score (Elixhauser index), patient’s neighborhood SES as measured by the ADI and English language preference. The Elixhauser index is a weighted score that considers a set of 30 comorbidities and their association with adverse outcomes such as in-hospital mortality and length of stay (13). The ADI uses factors such as income, education, employment, and housing quality to rank neighborhoods by socioeconomic disadvantage at a national or state level (11). For this study, we used national ADI ranks. Adjusted ORs with 95% confidence intervals (CIs) were reported. Missing data were present for several key variables, including race/ethnicity (22%), marital status (7.6%), insurance type (8.1%), comorbidity score (7.7%), area deprivation index (29.9%), and age at neurology consultation (4.9%). To address missingness, we used multiple imputation with five imputed datasets, incorporating all variables in the imputation model. Statistical significance was defined as a *p*-value < 0.05, and all analyses were conducted using R and SAS.

### Sensitivity analyses

We performed a sensitivity analysis by incorporating socioeconomic variables from the US Census Bureau, including median household income, poverty rate, racial composition, education levels, employment statistics, linguistic isolation, and household characteristics, retrieved using the tidycensus package in R. Estimates from the imputed datasets were combined using Rubin’s Rules to account for variability across imputations. Results were visualized using a forest plot to display adjusted ORs with 95% CIs. Additionally we completed a separate sensitivity analysis stratified by diagnosis, comparing those with PD and ET, excluding those with multiple diagnoses or unclear diagnosis (e.g., unspecified tremor). Results were summarized in a table.

## Results

A total of 3,286 patients with PD, ET, and other tremors were seen by movement disorders clinicians between 2012 and 2022 (Table 1). Of these, 397 (12.1%) underwent DBS surgery. Patients who underwent DBS were more likely to be younger (mean age 63.4 ± 10.7 years vs. 69.3 ± 11.2 years), male (63.8% vs. 36.2%), and had less comorbidities (Elixhauser Score 9 ± 8.7 vs. 11.5 ± 10.7) Although Hispanic patients in our clinic only comprised 6% of all PD, ET, and other tremor patients, there was a higher percentage of Hispanic patients in the surgical group (16.1% vs. 5.4%). Additionally, there was a higher percentage of individuals preferring a language other than English in the surgical group, compared to the non-surgical group (13.2% vs. 9.2%).

**Table 1:** Demographics of movement disorders patients and DBS completion rate (2012–2022).

Characteristic	Overall	DBS	No DBS	*P* value^b^
N	3,288	447	2,839	<0.0001
Age at initial visit, mean (SD)	68.55 (11.3)	63.4 (10.4)	69.3 (11.2)	
Sex, n (%)				0.1866
Female	1,284 (39.1)	162 (36.2)	1,122 (39.5)	
Male	2002 (60.9)	285 (63.8)	1717 (60.5)	
Diagnosis, *n* (%)^a^				
Essential tremor	722 (22.0)			
Parkinson’s disease	2,519 (76.6)			
Both	45 (1.4)			
Insurance, *n* (%)				0.0008
Commercial	1,546 (47.1)	168 (43.9)	1,378 (52.3)	
HMO	98 (3.0)	8 (2.1)	90 (3.4)	
Medicare	782 (23.8)	109 (28.5)	673 (25.5)	
Medi-Cal	287 (8.7)	55 (14.4)	232 (8.8)	
Other	306 (9.3)	43 (11.2)	263 (10.0)	
Did not specify	269 (8.2)	–	–	
Race/Ethnicity, *n* (%)				<0.0001
Non-Hispanic White	1,617 (49.2)	258 (57.7)	1,359 (47.9)	
Non-Hispanic, Other	501 (15.3)	64 (14.3)	437 (15.4)	
Hispanic	226 (6.9)	72 (16.1)	154 (5.4)	
Asian	218 (6.6)	26 (5.8)	192 (6.8)	
Did not specify	724 (22.0)	27 (6.0)	697 (24.6)	
Preferred language, n (%)^a^				0.0093
English	2,964 (90.2)	388 (86.80)	2,576 (90.74)	
Non-English	322 (9.8)	59 (13.20)	263 (9.26)	
Marital status, *n* (%)				<0.0001
Divorced/separated	290 (9.5)	52 (11.6)	238 (9.2)	
Married	2,104 (69.2)	300 (67.1)	1804 (69.6)	
Single	348 (11.4)	54 (12.1)	294 (11.3)	
Widowed	284 (9.3)	39 (8.7)	245 (10.4)	
Other	15 (0.5)	2 (0.5)	13 (0.5)	
Did not specify	247 (7.5)	–	–	
Area Deprivation Index, mean (SD)				<0.0001
National Rank	9.5 (12.8)	15.3 (16.7)	8.5 (11.8)	
Elixhauser Comorbidity Score, mean (SD)	11.1 (10.4)	9.0 (8.7)	11.5 (10.7)	<0.0001

a Missing data <1%.

b *p*-value for within-group comparison.

In terms of socioeconomic variables, there was a higher proportion of surgical patients on public insurance (28.5% Medicare and 14.4% Medi-Cal, compared to 25.5 and 8.8%, respectively, in the non-surgical group). Patients in the surgical group also lived in neighborhoods with lower SES, as measured by higher scores in the ADI (15.3 ± 16.7 vs. 8.5 ± 11.8).

### Adjusted logistic regression analysis

**Figure 1:**
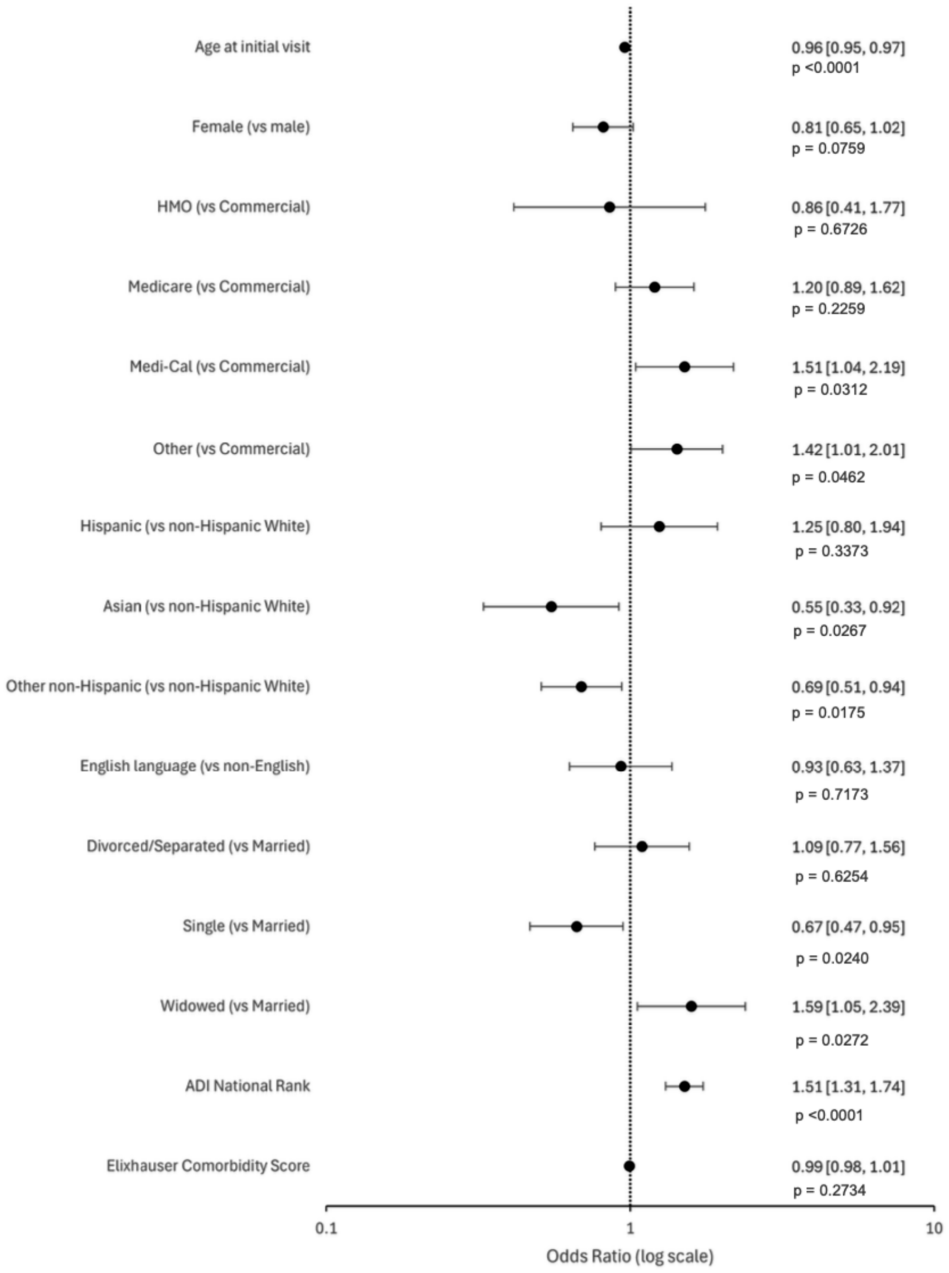
Forest plot of adjusted odds ratio of completing or not completing DBS surgery with imputed missing race/ethnicity and marital status data. Reference odds ratios (ORs) are accepted as 1 (or 10^0^ on log scale). OR values below 10^0^ estimate lower odds of DBS surgery, whereas values above 10^0^ serve as higher odds of DBS surgery. The 95% confidence intervals are given in parenthesis.

In adjusted analysis with imputations for missing race/ethnicity and unknown marital status (Figure 1, Table 2), increasing age was associated with lower odds of DBS (AOR 0.96, 95% CI 0.95–0.97, *p* < 0.001), as was female sex (AOR 0.81, 95% CI 0.65–1.02, *p* = 0.076), though the latter was not statistically significant.

**Table 2:** Logistic regression analysis showing the unadjusted odds ratio (OR) for DBS surgery implantation of patients referred for surgery (*n* = 3,286).

Variable	Unadjusted results			Model 1		
	OR	95% CI	*p*-value	AOR	95% CI	*p*-value
Age at initial visit	0.958	0.947–0.968	<0.0001	0.958	0.948–0.968	<0.0001
Sex						
Female	0.797	0.630–1.007	0.0577	0.814	0.648–1.022	0.0759
Male^^^	1	–	–	1	–	–
Insurance						
Commercial^^^	1	–	–	1	–	–
HMO	0.643	0.271–1.527	0.3217	0.855	0.414–1.766	0.6726
Medicare	1.457	1.093–1.941	0.0109	1.203	0.893–1.620	0.2259
Medi-Cal	1.433	0.959–2.141	0.0800	1.510	1.042–2.188	0.0312
Other	1.585	1.084–2.319	0.0177	1.423	1.007–2.010	0.0462
Race/Ethnicity						
Non-Hispanic White^^^	1	–	–	1	–	–
Hispanic	1.134	0.775–1.660	0.5181	1.247	0.802–1.938	0.3373
Asian	0.539	0.339–0.856	0.0088	0.550	0.329–0.918	0.0267
Non-Hispanic, Other	0.729	0.533–0.998	0.0484	0.690	0.509–0.937	0.0175
Preferred language						
English	0.859	0.577–1.278	0.4537	0.931	0.632–1.371	0.7173
Non-English^^^	1	–	–	1	–	–
Marital status						
Married^^^	1	–	–	1	–	–
Divorced/Separated	1.064	0.752–1.525	0.7370	1.093	0.765–1.563	0.6254
Single	0.660	0.460–0.947	0.0240	0.666	0.468–0.948	0.0240
Widowed	1.610	1.071–2.421	0.0222	1.588	1.054–2.391	0.0272
Other	0.192	0.086–0.430	<0.0001			
Area Deprivation Index						
National Rank	1.594	0.120–0.282	<0.0001	1.508	1.308–1.739	<0.0001
Elixhauser Comorbidity Score	0.986	0.974–0.998	<0.0001	0.993	0.981–1.005	0.2734

Model 1 represents the sensitivity analysis with race/ethnicity and marital status imputations (excluding *n* = 15 with unknown marital status) and adjusted OR. ^^^Reference variable.

Compared to non-Hispanic White patients, Asian patients had significantly lower adjusted odds of receiving DBS (AOR 0.55, 95% CI 0.33–0.92, *p* = 0.027), as did other non-White, non-Hispanic groups. Hispanic patients showed a trend towards higher odds of receiving DBS (AOR 1.25, 95% CI 0.80–1.94, *p* = 0.337) but this was not statistically significant. Regarding marital status, single individuals had lower adjusted odds of receiving DBS compared to those who were married (AOR 0.67, 95% CI 0.47–0.95, *p* = 0.0240), while widowed individuals had significantly higher odds compared to married patients (AOR 1.59, 95% CI 1.05–2.39, *p*-value = 0.272).

Compared to those with commercial insurance, California sponsored Medicaid patients (Medi-Cal) exhibited higher adjusted odds (AOR 1.51, 95% CI 1.04–2.19, *p* = 0.0312) of completing DBS surgery compared to those with commercial insurance, as well those with other non-commercial insurance (AOR 1.423, 95% CI 1.007–2.010, *p*-value = 0.0462). There was a trend towards higher odds in Medicare insurance though this was not statistically significant.

There was no statistically significant difference in odds of receiving surgery based on Elixhauser Comorbidity Score (AOR 0.99, 95% CI 0.98–1.01, *p*-value = 0.2734). Whereas higher ADI national rank (i.e., more disadvantaged) was associated with increased odds of DBS (AOR 1.51, 95% CI 1.31–1.74, *p*-value<0.0001).

### Sensitivity analyses

A sensitivity analysis using multiple imputations incorporating US Census-derived socioeconomic variables produced similar results, supporting the robustness of our findings (Table 3). An additional sensitivity analysis stratified by diagnosis of PD and ET additionally showed similar OR trends between diagnosis, justifying our combination of the diagnoses throughout other analyses (Supplementary Table 1). Though there were some differences noted between sex and insurance in PD compared to ET patients, the study was not powered to detect interactions across these variables due to the small sample size.

**Table 3:** Logistic regression model with multiple imputation for race/ethnicity and covariates using Census variables.

Variable	OR	95% CI	*p*-value
Age at initial visit	0.957	0.947–0.967	<0.001
Sex			
Female	0.794	0.632–0.997	0.0475
Male	1	–	–
Insurance			
Commercial	1	–	–
HMO	0.658	0.303–1.426	0.2888
Medicare	1.498	1.121–2.003	0.0073
Medi-Cal	1.507	1.004–2.264	0.0503
Other	1.645	1.121–2.416	0.0114
Race/ethnicity			
Non-Hispanic, White	1	–	–
Non-Hispanic, Other	0.776	0.569–1.059	0.1111
Hispanic	1.238	0.859–1.784	0.2536
Asian	0.615	0.386–0.979	0.0415
Preferred language			
English	0.828	0.559–1.228	0.3483
Non-English	1	–	–
Marital status			
Married	1	–	–
Divorced/separated	1.177	0.828–1.673	0.3639
Single	0.722	0.507–1.029	0.0719
Widowed	1.606	1.073–2.403	0.0212
Other	0.114	0.052–0.247	<0.0001
Area Deprivation Index			
National Rank	1.479	1.326–1.651	<0.0001
Elixhauser Comorbidity Score	0.990	0.978–1.003	<0.0001

## Discussion

Patients seen at our institution for routine movement disorders care differed from those who received DBS surgery in terms of age, insurance coverage, marital status, and ADI. When adjusting for all clinical and socioeconomic covariates, individuals with DBS within our center were more likely to be younger at initial evaluation, with California sponsored Medicaid coverage, widowed marital status, and with a higher socioeconomic disadvantage. Those of Asian ethnicity, other non-Hispanic ethnicity, and single marital status were less likely to be in the DBS group.

### Sex differences

Several studies conducted in various countries have overwhelmingly found that women, with either PD or ET, are less likely to undergo DBS surgery as compared to men (2, 5, 7, 14, 15). Similar to other studies, women were underrepresented in the surgical group (36.2% vs. 63.8%). Though our study did not identify a significant sex difference among those who did and did not complete surgery (AOR 0.81, 95% CI 0.65–1.02, *p* = 0.076), there was a trend towards lower odds. One possible explanation for the lack of significant sex-based differences is the increased likelihood of gender-concordant care at our center given the high proportion of female Movement Disorders physicians who were practicing at the time of the study. While we did not distinguish the referring Movement Disorders physician and patient outcome, this would be of interest in future research to establish if gender-concordant care facilitates increased odds of surgery as noted in other studies (2).

### Race and ethnicity

Our single-center results suggest that, compared to national trends in DBS surgery and when adjusting for various clinical and socioeconomic covariates, there are no statistically significant differences in the odds of receiving DBS surgery for Hispanic individuals, though Asian patients had lower odds of DBS surgery (3). Previous studies have not shown disparities between Asian and White patients, with the exception of one paper that grouped Asians with other minority groups and did not include a separate analysis (3–9, 16). Another study have found that racial disparities in minoritized groups may be mediated by proximity to a DBS center, with those living closer to DBS centers being more likely to undergo surgery, and those living farther less likely, with a large proportion of minoritized patients living farther from these centers (17). However, there are limited studies specific to DBS surgery patterns among Asian American.

In terms of our Hispanic patient findings, it is possible we did not find a difference in odds of DBS given our institution’s availability of neuropsychological assessment through the Hispanic Neuropsychiatric Center of Excellence, which may alleviate a potential cultural and language barrier to proceeding with surgery (18). This is further supported as non-English preference did not significantly change odds of surgery. Another explanation for the similar odds of surgery for Hispanic and non-Hispanic White people may be that Hispanic individuals referred to our center for surgery are simply presenting later in the disease cycle with more severe symptoms and in turn more likely to be appropriate candidates for surgery. While the similar odds for Hispanic patients receiving DBS surgery at this center provide cautious optimism about the equitable care provided at this center, further investigation is needed to fully understand how we are serving this community.

Given the small number of African American or Black patients (*n* = 42, approximately 1.28% of total patients analyzed) seen at our center, we did not analyze this group in multivariate analysis. However, this finding raises a significant disparity for this patient group as it is underrepresented in our center relative to LA County demographics, which is 69% White, 9% Black, and 16% Asian, with 49% of residents identifying as Hispanic or Latino (10). Our findings highlight the importance of evaluating referral and access patterns in DBS research, as underrepresentation at the referral level can mask downstream disparities in surgical care.

### Marital status

In our study, single individuals were less likely to receive DBS surgery as compared to married individuals (AOR 0.67, 95% CI 0.47–0.95, *p* = 0.0240). While an indirect measure, marital status may serve as a surrogate marker for level of social support which has been identified as an important factor considered during the preoperative period (2, 19, 20). It is not clear if low social support contributes to referral bias or if it is associated with other comorbid features that make one a poor surgical candidate. For example, collateral information from caregivers may highlight cognitive decline or behavioral issues that the patient may not recognize (20). Interestingly, widowed individuals had higher odds of DBS surgery (AOR 1.59, 95% CI 1.05–2.39, *p*-value = 0.272) which may suggest an ongoing immediate support network that single individuals may not have access to. Furthermore, the interaction between gender and social support is an important area of future research as this has been suggested as a potential barrier for women undergoing surgical evaluation (19).

### Socioeconomic status and insurance

Compared to high SES and privately insured individuals, patients from low SES backgrounds and those with Medicare had a higher likelihood of completing DBS surgery. As our center accepts both internal and external referrals for DBS surgery, this may reflect a referral bias in which complex low-SES patients are more likely to be referred for advanced therapies (i.e., DBS) and less likely to be referred for routine care. On the other hand, patients in earlier disease stages, who are more likely to be privately insured and from higher SES backgrounds may access routine care at an academic institution more often. These findings are consistent with the ADI results which showed that those from areas with high ADI were more likely to undergo DBS. While these findings are reassuring from a surgical standpoint within the health system, they highlight disparities in access to general movement disorders care. Groups from financially disadvantaged backgrounds appear less likely to be seen at our center for non-surgical care and more likely to present with complex disease requiring DBS.

We also found that Medicare patients had a higher odds of DBS surgery. This is counterintuitive considering that older age was less associated with DBS surgery. Given that Medicare eligibility is based on age (65 and older), as well as evidence of a qualifying disability, it is likely Medicare beneficiaries who received DBS in our clinic were those eligible for Medicare based on disease severity and disability (particularly in the case of PD), and less so based on age eligibility. These findings also highlight a possible referral bias with Medicare beneficiaries with a qualifying disability (such as advanced PD), being more likely to be seen in our center for DBS consideration, even if indeed younger in age. In contrast, self-referred patients with private insurance may have less need for DBS, due to lack of screening Nationally, Medicare patients consist of the highest block of patients receiving DBS (86% for PD, 77% for ET), but are less likely to receive DBS for PD and equally likely to receive DBS for ET when compared to privately insured patients by odds ratios (3). These data further suggest that the increased OR for DBS for Medicare patients at this single institution in LA County are likely due to increased disease severity at time of referral. However, analysis of disease severity by insurance type would be of interest in future investigations into DBS access at our center and beyond.

### Limitations

This is a single-institution retrospective study of a large metropolitan center in Southern California. Therefore, our findings may not be generalizable to other healthcare systems outside the US. However the methodology used for this study in assessing equitable access to DBS surgery may still be reproducible in other healthcare systems at both an institutional and regional level. Additionally, a center-specific assessment of DBS outcomes is likely to ensure tailored and effective approaches that improve access to DBS care in a specific region. Due to the retrospective and non-randomized study design we were not able to account for all confounding variables and future larger studies controlling for these variable may help to strengthen the analysis. Regarding racial and ethnic disparities, we also interpret these results with caution, considering the relatively small sample size of Hispanic (6.9%) and Asian patients (6.7%) seen at our center, which may underestimate potential racial and ethnic disparities. Furthermore, although our Hispanic cohort was larger than in previous studies, Hispanic, Black, and Asian patients remain underrepresented relative to local demographics (10). Other important sociodemographics variables have been identified as influencing DBS completion (2, 7–9) such as caregiver support, health literacy, and cultural beliefs, however these were not able to be captured due to the limitations of an EMR-based structured data query. Despite these limitations, this study has several strengths, including its analysis of a racially and socioeconomically diverse cohort in a large metropolitan center. Future research will more closely examine each racial and ethnic cohort seen at our center to better identify system-, provider- and patient-level barriers that influence surgical decisions. Though our study did not include patients with other movement disorders (such as dystonia) due to concerns for heterogeneity, future dedicated analysis of this group may help identify sociodemographic factors in DBS utilization unique to this cohort.

## Conclusion

Our study at a large tertiary center in LA County, suggests that differences in completion of DBS surgery for movement disorders are mediated by differences in referrals and access to general movement disorders care within our center. Whereas we had encouraging findings regarding DBS surgery among low SES and Medicare beneficiaries, our study also finds potential gaps in equitable care. Future qualitative studies should examine the role of cultural values in interest in DBS surgery among historically minoritized groups, as well as provider level attitudes regarding DBS referrals, including general neurologists. Additionally, future mixed methods studies leveraging the use of EHR bioinformatics, qualitative research studies, and community engagement are needed to (1) understand referral pathways for various groups and (2) draft appropriate multilevel interventions to ensure all movement disorders patients have equitable access to routine movement disorders care and advanced surgical interventions such as DBS.

## Data Availability

The original contributions presented in the study are included in the article/Supplementary material, further inquiries can be directed to the corresponding author.
