# Supplementary material for: Access to movement disorders care and advanced surgical therapies in a tertiary care center
Source: Front Neurol. 2026 Mar 31;17:1743834. doi: 10.3389/fneur.2026.1743834 (PMC13076168; doi:10.3389/fneur.2026.1743834)
Supplement: Supplementary file 1 [file Table_1.pdf]

SUPPLEMENTAL MATERIALS

SUPPLEMENTAL TABLE 1. Sensitivity analysis stratified by diagnosis of Parkinson's Disease (PD) or Essential Tremor (ET).

Variable	ET OR	ET 95% CI	ET P-Value	PD OR	PD 95% CI	PD P-Value
**Age at initial visit**	0.987	0.969–1.006	0.183	0.943	0.930–0.956	<.0001
**Sex**						
**Female**	1.043	0.668–1.629	0.854	0.739	0.555–0.983	0.038
**Male**	1			1		
**Insurance**						
**Commercial**	1			1		
**HMO**	1.923	0.530–6.972	0.321	0.768	0.321–1.833	0.552
**Medicare**	1.616	0.887–2.945	0.119	1.148	0.828–1.591	0.408
**Medi-Cal**	1.492	0.673–3.312	0.325	1.546	0.980–2.439	0.065
**Other**	1.668	0.863–3.225	0.129	1.283	0.809–2.034	0.293
**Race/Ethnicity**						
**Non-Hispanic, White**	1			1		
**Non-Hispanic, Other**	0.592	0.319–1.099	0.097	0.713	0.492–1.032	0.073
**Hispanic**	1.084	0.435–2.697	0.863	1.149	0.734–1.799	0.543
**Asian**	0.181	0.023–1.438	0.106	0.602	0.367–0.988	0.045
**Unknown race or ethnicity**	0.216	0.109–0.430	<.0001	0.113	0.065–0.196	<.0001
**Preferred language**						
**English**	1.154	0.422–3.158	0.78	0.87	0.562–1.348	0.534
**Non-English**	1			1		
**Marital status**						
**Married**	1			1		
**Divorced/Separated**	0.873	0.418–1.822	0.717	1.057	0.697–1.602	0.793
**Single**	0.953	0.496–1.829	0.884	0.561	0.359–0.876	0.011
**Widowed**	1.206	0.586–2.484	0.611	1.725	1.036–2.873	0.036
**Area Deprivation Index**						
**National Rank**	1.503	1.233–1.832	<.0001	1.555	1.342–1.801	<.0001
**Elixhauser Comorbidity Score**	0.983	0.962–1.004	0.11	0.99	0.975–1.005	0.188
